# Changes in cross-sectional areas of posterior extensor muscles in thoracic spine: a 10-year longitudinal MRI study

**DOI:** 10.1038/s41598-022-19000-2

**Published:** 2022-08-30

**Authors:** Hitoshi Umezawa, Kenshi Daimon, Hirokazu Fujiwara, Yuji Nishiwaki, Takehiro Michikawa, Eijiro Okada, Kenya Nojiri, Masahiko Watanabe, Hiroyuki Katoh, Kentaro Shimizu, Hiroko Ishihama, Nobuyuki Fujita, Takashi Tsuji, Masaya Nakamura, Morio Matsumoto, Kota Watanabe

**Affiliations:** 1 Department of Orthopedic Surgery, Keio University School of Medicine, 35 Shinanomachi, Shinjuku-ku, Tokyo Japan; 2 Department of Diagnostic Radiology, Keio University School of Medicine, 35 Shinanomachi, Shinjuku-ku, Tokyo Japan; 3 Department of Environmental and Occupational Health, School of Medicine, Toho University, 5-21-16 Omorinishi, Ota-ku, Tokyo Japan; 4 Department of Orthopedic Surgery, Isehara Kyodo Hospital, 345 Tanaka, Isehara-shi, Kanagawa Japan; 5 Department of Orthopedic Surgery, Tokai University School of Medicine, 143 Shimokasuya, Isehara-shi, Kanagawa Japan; 6 Department of Orthopedic Surgery, Spine Center, Sanokosei General Hospital, 1728 Horigomecho, Sano-shi, Tochigi Japan; 7 Fujita Health University Orthopedic Surgery, 1-98 Kutsukakecho, Toyoake-shi, Aichi Japan; 8 National Hospital Organization Tokyo Medical Center, 2-5-1 Higashigaoka, Meguro-ku, Tokyo Japan

**Keywords:** Musculoskeletal system, Disease prevention, Geriatrics, Health policy, Medical imaging, Patient education, Anatomy, Health care, Risk factors, Signs and symptoms, Medical research, Epidemiology

## Abstract

Age-related changes in the posterior extensor muscles of the cervical and lumbar spine have been reported in some studies; however, longitudinal changes in the thoracic spine of healthy subjects are rarely reported. Therefore, this study aimed to evaluate changes in the cross-sectional areas (CSAs) of posterior extensor muscles in the thoracic spine over 10 years and identify related factors. The subjects of this study were 85 volunteers (mean age: 44.7 ± 11.5) and the average follow-up period was about 10 years. The CSAs of the transversospinalis muscles, erector spinae muscles, and total CSAs of the extensor muscles from T1/2 to T11/12 were measured on magnetic resonance imaging. The extent of muscle fat infiltration was assessed by the signal intensity (luminance) of the extensor muscles’ total cross-section compared to a section of pure muscle. We applied a Poisson regression model, which is included in the generalized linear model, and first examined the univariate (crude) association between each relevant factor (age, sex, body mass index, lifestyle, back pain, neck pain, neck stiffness, and intervertebral disc degeneration) and CSA changes. Then, we constructed a multivariate model, which included age, sex, and related factors in the univariate analysis. The mean CSAs of the transversospinalis muscles, erector spinae muscles, and total CSAs of the extensor muscles significantly increased over 10 years. Exercise habit was associated with increased CSAs of the erector spinae muscles and the total area of the extensor muscles. The cross-section mean luminance significantly increased from baseline, indicating a significant increase of fat infiltration in the posterior extensor muscles. Progression of disc degeneration was inversely associated with increased fat infiltration in the total extensor muscles.

## Introduction

The posterior extensor muscles of the thoracic spine help maintain sagittal alignment and facilitate spinal motion, including dorsiflexion and rotation of the thoracic spine^1,2^. Dysfunction or atrophy of these muscles can result in malalignment of the thoracic spine and a reduced range of motion, significantly affecting activities of daily living^3,4^. Sasaki^5^ conducted a cross-sectional study that measured the cross-sectional areas (CSAs) of lumbar paraspinal muscles in asymptomatic populations using axial T2-weighted magnetic resonance imaging (MRI). The CSAs of the erector spinae, multifidus, and psoas muscles were found to be generally larger in males than in females and decreased with age. Fat infiltration of the posterior extensor muscles is generally higher in females than in males and has been reported to be associated with disuse, altered leptin signaling, sex steroid deficiency, and exposure to glucocorticoids^6,7^. Although several studies have reported age-related changes in the posterior extensor muscles of the cervical and lumbar spines^8–14^, longitudinal changes in the thoracic spine of healthy subjects are rarely reported^15^. The present study aimed to evaluate the 10-year longitudinal changes in the CSAs of the thoracic posterior extensor muscles using MRI and to investigate whether these changes were associated with clinical symptoms and lifestyle habits.

## Methods

### Ethical statement

This study was approved by the ethics committee of Keio University School of Medicine (approval number 20150050), and written informed consent was obtained from all participants. Experiments were conducted following the Ministry of Education, Culture, Sports, Science, and Technology of Japan, and Ministry of Health, Labor, and Welfare of Japanese guidelines.

### Subjects

**Figure 1:**
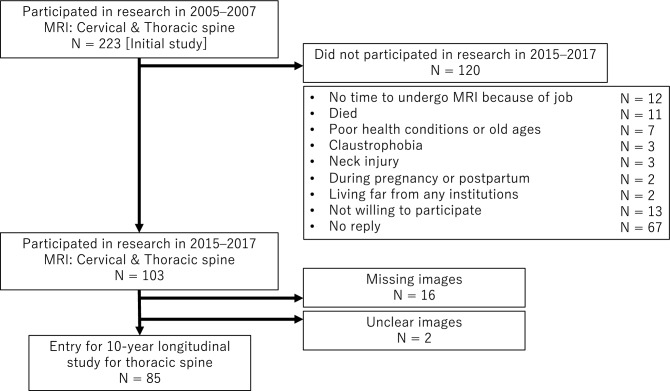
Study design. This study evaluated the 10-year longitudinal changes of the paravertebral muscles in the thoracic spine and was conducted as part of a 20-year longitudinal study of the cervical spine.

The present study, which examines the 10-year longitudinal changes of the thoracic paravertebral muscles, was conducted as part of a 20-year longitudinal MRI study of the cervical spine^8–12^ (Fig. 1). Whole-spine MRI images were obtained from a total of 223 asymptomatic subjects during the period from 2005 to 2007 in the initial study. For this study, we contacted the original subjects by mail and requested their participation in a 10-year follow-up study taking place between 2015 and 2017. Among the 223 original subjects, 103 agreed to participate in the present study (follow-up rate of 46.2%), and MRI and physical examinations were conducted at eight of the original eleven participating institutions. The remaining 120 volunteers could not be followed up for the thoracic MRI investigation. The omission of the required imaging sequences or the lack of image quality that allowed for clear visualization of the borders of the posterior extensor muscle led to the exclusion of 18 cases, leaving 85 cases as the subjects of this study (final follow-up rate of 38.1%) (Fig. 1). These 85 subjects comprised 36 office workers, 35 healthcare workers (doctors, nurses, and medical coworkers), 11 manual workers (construction and farming), and 13 others (housewives and retired individuals).

There were 50 males and 35 females subjects, with a mean age of 44.7 ± 11.5 years at the initial study conducted 10 years ago (distribution shown in Table 1) and a mean interval of 9.9 ± 0.8 years between the MRI studies. The remaining 138 were volunteers, comprising 74 males and 64 females, with a mean age of 55.7 ± 15.6 years. All subjects completed questionnaires related to clinical symptoms and lifestyle habits including alcohol (regularly consumed alcohol during 10-year period) and exercise habit (regular participation in a exercise at least 1 h once a week) and underwent physical examinations by spine surgeons.

**Table 1:** Profiles of 85 subjects at initial study.

Mean age (years)	44.7 ± 11.5
Age distribution (%)	Number (%)
≤ 20–29	6 (7.1)
30–39	28 (32.9)
40–49	28 (32.9)
50–59	11 (13.0)
≥ 60	12 (14.1)
**Sex (%)**	
Male	50 (58.9)
Female	35 (41.1)
**Body mass index (%)**	
< 25	65 (76.5)
≥ 25	20 (23.5)

### MRI examination

In the initial study, MRI were acquired using a 1.5-Tesla (T) (SIGNA Excite HD 1.5T, General Electronic, WI, USA) superconducting MRI scanner, following the imaging protocol described in our earlier report^9^. In the present study, at a principal institution where approximately half of the subjects were examined by MRI, MR images were captured using a fast spin-echo technique using a 1.5T superconducting scanner and phased array coils (SIGNA; GE Healthcare) with the following sequence: a T2-weighted axial images. The protocol used at other institutions was the same as the one used at the principal institution, i.e., a fast spin-echo sequence. T2-weighted axial images parallel to each vertebral discs were analyzed in this study. The imaging protocols at the participating institutions are shown in Supplementary 1.

### Measurements of paraspinal muscle cross-sectional area

**Figure 2:**
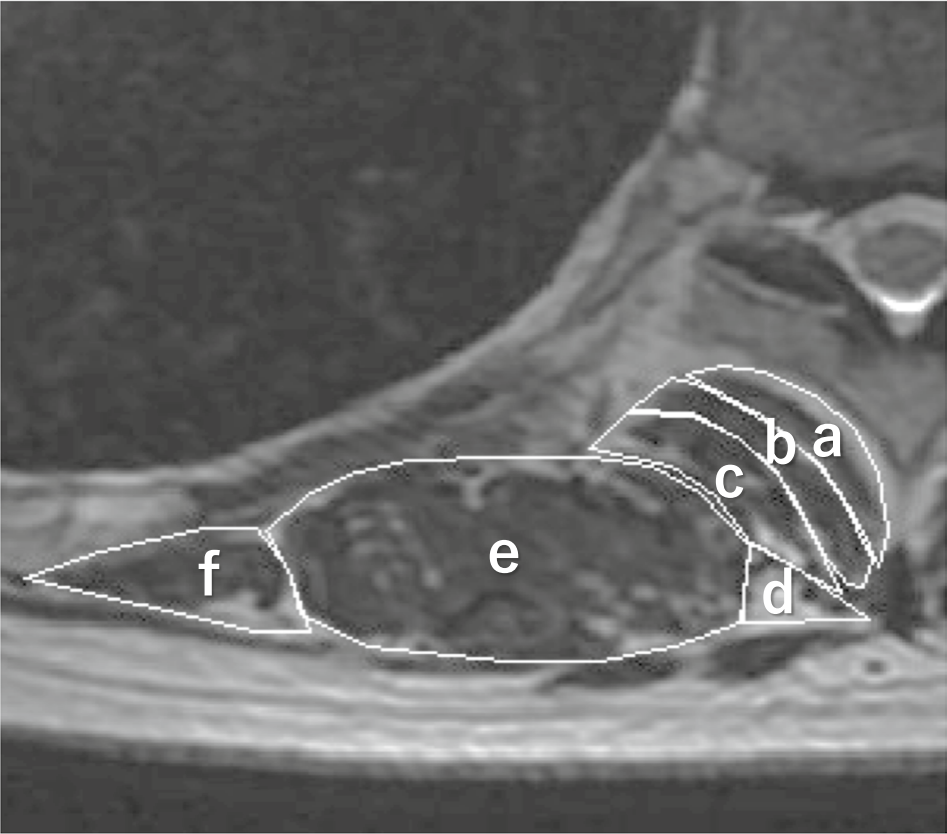
Posterior extensor muscles visualized on an axial T2-weighted image. The cross-sectional area of the transversospinalis muscles (a, rotatores; b, multifidus; c, semispinalis), erector spinae muscles (d, spinalis; e, longissimus; f, iliocostalis) were measured by using the calibrated scale on the MR images.

The CSAs of the transversospinalis muscles (rotatores, multifidus, semispinalis), erector spinae muscles (spinalis, longissimus, iliocostalis), and total extensor muscles at each thoracic intervertebral level were measured on T2-weighted axial images after scale calibration. Using Image J 1.52a, a Java-based version of the public domain NIH software, the fascial border of each muscle was manually traced (Fig. 2), yielding the area of the muscle cross-section. Manual tracing was performed following the protocol by Pai et al.^15^ Each measurement was taken twice, and the values were averaged.

The measurements for each thoracic intervertebral level from T1/2 to T11/12 were compared between the initial and 10-year follow-up MRI studies. The change in muscle CSAs was calculated as follows:

$${\text{CSAs\,change}}\,\,{{(\% )}} = {\text{(CSAs\,at\,follow-up}} - {\text{CSAs\,at\,initial\,study)/CSAs\,at\,initial\,study}}\, \times \,100.$$

The CSAs used in this study are anatomical CSAs that change with postural and muscle activation and are different from physiological CSAs that describe the ability of muscles to generate force.

### Evaluation of fat infiltration of the muscle

The degree of fat infiltration was evaluated by the luminance of the cross-section of each extensor muscle group at each thoracic intervertebral level, using the luminance of pure muscle tissue as reference. Since the luminance of fat tissue is higher than muscle, increased fat infiltration of muscle will be reflected as increased luminance. Fat infiltration rate was calculated as follows:^16,17^

$${\text{Fat\,infiltration\,rate}}\,{{(\% )}}\, = \,{\text{luminance\,in\,CSAs\,of\,whole\,muscle/luminance\,in\,pure\,muscle\,luminance}}\, \times \,100.$$

The changes in the fat infiltration rate were compared between the initial and follow-up study.

### Evaluation of intervertebral disc degeneration

Degeneration of the intervertebral disc was evaluated based on the following five findings on MRI: (1) decrease in the signal intensity of the intervertebral discs, (2) anterior compression of the dura and the spinal cord, (3) posterior disc protrusion, (4) disc-space narrowing, and (5) foraminal stenosis. MR findings were scored with the Matsumoto classification^8^. The scoring system for the different MR findings is shown in Supplementary 2. An increase of at least 1 grade in any intervertebral level was considered to be the progress of degeneration. Two experienced neuroradiologists blinded to the subjects independently read the MRI images and graded the intervertebral discs. The final results used the findings of one of the two neuroradiologists. The interobserver reliability was good to excellent, as reported previously^10^.

### Statistical analysis

Student's *t* tests were used to compare the mean CSAs of the study muscles in the initial study with those in the 10-year follow-up study. We applied a Poisson regression model, which is included in the generalized linear model, and first examined the univariate (crude) association between each relevant factor (age, sex, medical history, lifestyle, and disc degeneration) and CSA changes. Then, we constructed a multivariate model, which included age, sex, and related factors in univariate analysis.

A *P* value of < 0.05 was considered to be indicative of statistical significance. All statistical analyses were performed using Dr. SPSS II for Windows (SPSS Japan Inc., Tokyo, Japan) and Stata15 for Windows (Stata Corporation, College Station, TX, USA).

Inter- and intraobserver errors were evaluated to measure the paraspinal muscle CSA. For the interobserver error assessment using ICC (2, 1), 20 randomly chosen MR images were measured independently by the first (H.U.) and second (H.I.) investigators. For the intraobserver error assessment using ICC (1, 2), 20 images were randomly selected, and images were measured 2 times with a 3-month interval. The values measured by the first investigator (H.U.) were used in this study.

## Results

### Changes in cross-sectional area over 10 years

**Figure 3:**
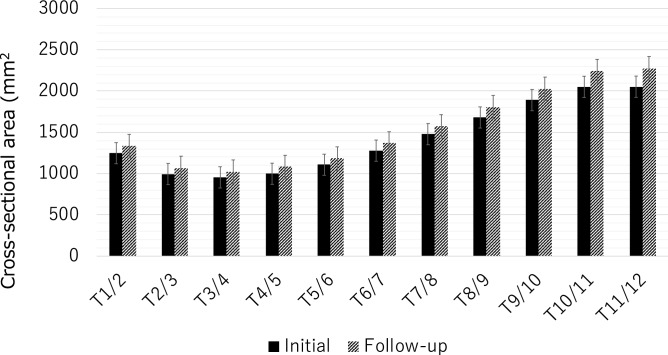
The cross-sectional areas of the total posterior extensor muscles at each intervertebral disc level. The cross-sectional areas of the total posterior extensor muscles significantly increased over 10 years at all levels from T1/2 to T11/12.

The mean CSAs of the transversospinalis muscles, erector spinae muscles, and combined extensor muscles significantly increased over 10 years, from 557 ± 105 to 609 ± 119 mm^2^, from 873 ± 225 to 934 ± 234 mm^2^, and from 1430 ± 321 to 1543 ± 342 mm^2^, respectively, by Student’s *t*-test. The CSAs of each muscle group separately or combined significantly increased over 10 years at all intervertebral levels from T1/2 to T11/12 (Fig. 3). Regarding the intraobserver error in image evaluation, the estimated intraobserver error was 0.90 and the interobserver error was 0.87. Therefore, a good agreement was observed between these independent findings.

### Factors associated with changes in the cross-sectional areas of the muscles

Univariate analysis showed that exercise performed at least once a week was significantly associated with the changes in the CSAs of the erector spinae muscles and combined extensor muscles. The clinical symptoms (low back pain, neck pain, stiff shoulder), age, sex, smoking, alcohol, obesity, and progression of disc degeneration were not significantly associated with CSAs changes (Table 2).

**Table 2:** Univariate association between changes in CSA over ten years and factors tested in the Poisson regression model.

	Transversospinalis muscles		Erector spinae muscles		Total muscles	
	Relative risk (95% CI)	*P* value	Relative risk (95% CI)	*P* value	Relative risk (95% CI)	*P* value
Age (years)	0.9 (0.8–1.1)	0.23	0.8 (0.7–1.0)	0.10	0.8 (0.7–1.0)	0.06
Sex	1.2 (1.0–1.4)	0.051	1.2 (0.9–1.4)	0.23	1.1 (0.9–1.3)	0.49
Exercise habit	1.1 (0.9–1.3)	0.30	1.3 (1.1–1.7)	< 0.01*	1.2 (1.0–1.5)	0.02*
Body mass index (kg/m^2^)	1.1 (1.0–1.3)	0.08	0.9 (0.7–1.2)	0.48	1.0 (0.8–1.2)	0.76
Disc degeneration	1.0 (0.8–1.2)	0.88	0.9 (0.7–1.2)	0.56	0.9 (0.8–1.1)	0.42
Smoking	0.9 (0.7–1.3)	0.71	0.9 (0.6–1.4)	0.77	1.0 (0.7–1.3)	0.96
Alcohol	0.9 (0.8–1.1)	0.43	1.2 (0.9–1.5)	0.26	1.0 (0.8–1.2)	0.76
**Clinical symptoms**						
Low back pain	1.0 (0.9–1.2)	0.60	0.9 (0.7–1.1)	0.32	0.9 (0.8–1.2)	0.58
Neck pain	1.1 (1.0–1.3)	0.15	1.1 (0.8–1.4)	0.48	1.1 (0.9–1.3)	0.42
Stiff shoulder	1.1 (0.9–1.3)	0.30	1.0 (0.8–1.3)	0.83	1.1 (0.9–1.4)	0.24

Asterisks indicate statistical significance.

95% CI, 95% confidence interval.

The results of multivariate analysis are presented. CSAs of transversospinalis showed a significant increase in females with a relative risk (RR) of 1.2 (95% CI 1.0–1.4). The CSAs of erecter spinae muscles significantly increased the exercise habits with an RR of 1.4 (95% CI 1.1–1.7). The CSAs of the total muscles also significantly increased the exercise habits with an RR of 1.3 (95% CI 1.1–1.5) and showed a significant decrease at the age of > 40 years with an RR of 0.8 (95% CI 0.7–1.0) (Table 3).

**Table 3:** Multi-variable adjusted association between changes in CSA over ten years and factors in the Poisson regression model.

	Transversospinalis muscles		Erector spinae muscles		Total muscles	
	Relative risk (95% CI)	*P* value	Relative risk (95% CI)	*P* value	Relative risk (95% CI)	*P* value
Age (years)	0.9 (0.8–1.1)	0.21	0.8 (0.6–1.0)	0.07	0.8 (0.7–1.0)	0.048*
Sex	1.2 (1.0–1.4)	0.04*	1.2 (0.9–1.5)	0.14	1.1 (0.9–1.3)	0.36
Exercise habit	1.1 (0.1–1.2)	0.23	1.4 (1.1–1.7)	< 0.01*	1.3 (1.1–1.5)	0.01*

An asterisk indicates statistical significance.

95% CI, 95% confidence interval.

### Factors associated with changes in fat infiltration of total muscles over 10 years

The mean luminance of the posterior extensor muscles significantly increased from 121% in the initial study to 154% in the follow-up study. Univariate analysis revealed that the progression of disc degeneration was significantly associated with the changes in luminance over 10 years with an RR of 0.5 (95% CI 0.3–0.96). (Table 4). After adjusting for age, sex, and exercise habit using Poisson regression analysis, we revealed that progression of disc degeneration was negatively associated with increased luminance, with an RR of 0.5 (95% CI 0.3–0.97) (Table 5).

**Table 4:** Relationship between changes in the luminance of increased CSAs and factors evaluated using univariate analyses.

	Relative risk (95% CI)	*P* value
Age (years)	1.2 (0.6–2.1)	0.65
Sex	1.0 (0.5–1.7)	0.87
Body mass index (kg/m^2^)	0.8 (0.4–1.7)	0.59
Exercise habit	1.0 (0.5–1.8)	0.89
Smoking	1.0 (0.4–2.4)	0.94
Alcohol	0.9 (0.5–1.6)	0.67
Disc degeneration	0.5 (0.3–0.96)	0.04*
**Clinical symptoms**		
Low back pain	1.0 (0.6–1.8)	1
Neck pain	0.8 (0.4–1.8)	0.59
Stiff shoulder	1.0 (0.6–1.8)	0.96

An asterisk indicates statistical significance.

95% CI, 95% confidence interval.

**Table 5:** Relationship between changes in the luminance of increased CSAs and factors tested by Poisson regression analyses.

	Relative risk (95% CI)	*P* value
Age (years)	1.1 (0.6–2.0)	0.72
Sex	1.0 (0.6–1.8)	1.0
Exercise habit	1.0 (0.5–1.7)	0.9
Disc degeneration	0.5 (0.3–0.97)	0.04*

An asterisk indicates statistical significance.

95% CI, 95% confidence interval.

## Discussion

### Changes in the cross-sectional areas of the posterior extensor muscles over 10 years

In this 10-year longitudinal study, the CSAs of the posterior extensor muscles of the thoracic spine, including the transversospinalis muscles and erector spinae muscles, both separately and combined, significantly increased over 10 years at all intervertebral levels. Regarding the cervical spine, Okada reported that the CSAs of posterior extensor muscles in the cervical spine increased in patients in their teens up to 40 years old, but began to decrease after that^9^. Kennis conducted a 9.5-year longitudinal study using dual-energy X-ray absorptiometry and reported that whole-body muscle mass in middle-aged (35–39 years) men significantly increased^18^. In a study measuring skeletal muscle mass in 433 volunteers, Kyle reported that appendicular skeletal muscle mass peaked between the ages of 35 and 59 years in men, suggesting that age-associated muscle atrophy starts at the age of 50 or 60 years^19^. Janssen used whole-body MRI to assess the skeletal muscle in 468 males and females. They reported that the decline in skeletal muscles begins after the age of 50 years, regardless of the gender^20^. In the present study, the average age of participants was 44.7 ± 11.5 years, with 73% aged < 50 years in the initial study, and the proportion of males was 59% (Table 1). The average age of participants was one of the factors explaining the overall increase in the mean CSAs observed in this study. These results also suggest that the decrease in thoracic spine muscles is observed later than that in the whole-body and cervical spine muscles.

### Factors associated with the changes in the cross-sectional areas of muscles

Age of > 40 years was a significant factor associated with decreased CSAs of the posterior extensor muscles. In a previous study, Okada et al. reported that the CSAs of the posterior extensor of the cervical spine increased from their tens to thirties and decreased in the forties and later^9^. Furthermore, Cohn reported that an age-related decrease in muscle volume starts in the fifth or sixth decade of life^21^. Janssen reported that skeletal muscle mass began to decrease after the age of 50 years and that decreased skeletal muscle mass was significantly greater in males than in females^20^. In this study, CSAs of transversospinalis showed a significant increase in females. This result indicated that gender difference in the CSAs of transversospinalis there may be a characteristic of CSAs changes in thoracic spine.

In addition to age, exercising at least once a week at the time of follow-up was also a significant factor associated with the increase in the CSAs of the posterior extensor muscles. Several studies have reported increased muscle mass with exercise^22–27^. Franchi measured the CSAs of femur muscles in healthy subjects using MRI and reported a significant increase with exercise^23^. In a study of exercise and low back pain, Ko reported that exercise significantly increased the muscle strength of the posterior extensor muscles of the lower back^22^. Nutrition has also been reported to affect muscle mass^24–27^, but the present study did not collect information about nutrition status.

### Factors associated with changes in fat infiltration of total muscles over 10 years

In the present study, we compared the luminance of a muscle’s cross-section to the luminance of pure muscle to evaluate fat infiltration. The method used was previously reported by Fischer and Mengirardi, who evaluated the fat infiltration of muscle with magnetic resonance spectroscopy (MRS)^16,17^ by comparing the luminance of the entire muscle to a section of muscle without fatty degeneration. By analyzing the spectroscopy graphs, this method enables separate quantification of the muscular intra- and extracellular fat. Urrutia measured the CSA and fat signal fraction of intervertebral disc degeneration and erector spinae muscles (erector spinae and multifidus muscles) from L1–L2 to L5–S1. This increase was reportedly associated with an increase in fat signal fraction^7^. However, the results of this study showed that an increase in intervertebral disc degeneration was associated with a decrease in fat signal luminance. This is because Urrutia used the Pfirrmann classification to evaluate intervertebral disc degeneration, whereas this study used the Matsumoto classification, and the survey target site is different between the thoracic and lumbar spines. The Matsumoto scores were determined using decreased signal intensity of the intervertebral disc, anterior compression of the dura and spinal cord, posterior disc protrusion, disc-space narrowing, and foraminal stenosis. The Matsumoto scores may be useful in relatively easily assessing disc degeneration, including disc condition and structural disorders. The challenge is to improve the evaluation accuracy by further increasing the number of cases and continuing investigations.

### Study limitations

There are several limitations in this study that should be considered when interpreting our results. First, we measured muscle volumes only on T2-weighted axial images because axial images were acquired only with a T2-weighted pulse sequence in the initial trial protocol. Previous reports have investigated muscle structure by using T1-weighted^28–31^ or T2-weighted^32–34^ images, and the superiority of either sequence in illustrating the muscle structure has remained controversial. Moreover, CSAs measured in this study indicate anatomical CSA, which is different from the physiological CSA, representing the ability to generate muscle force. Therefore, it should be noted that the CSA measured in this study does not represent clinical muscle force. It is necessary to associate the results of this study with the physiological CSA that represent muscle force in future studies. Second, in this study, 1.5-T superconducting imagers and a fast spin-echo sequence were used. However, the types of MR imagers and software were not identical between the two testing periods, which might have resulted in some differences in the quality of images between the initial and follow-up MRI. To minimize the influence of differences in MRI conditions, we ensured to maintain consistency in image evaluations by following the same protocol as the one used at the principal institution, e.g., by grading the signal intensity of the muscle mass with reference to the cerebrospinal fluid intensity at the same level. However, the estimated intraclass correlation between the two investigators that was performed to assess the reliability and reproducibility of the measurements, showed good agreement. Third, the follow-up rate was not high (38.1%), indicating the possibility of selection bias. The subjects who could not be followed up were older than those who were followed up. This difference in age may have affected the results, particularly CSA changes on MRI. Therefore, this result cannot be applied to all age groups. Thus, no significant differences were observed in CSA changes by age group or gender. However, it is believed that more accurate results will be achieved in future studies by further increasing the age and number of cases.

Nonetheless, to the best of our knowledge, any longitudinal studies evaluating the changes in the posterior extensor muscles of the thoracic spine have not yet been conducted in healthy individuals. This paper is the first 10-year long-term follow-up study of the changes in thoracic spine and has critical clinical importance. Further, much attention has been focused on the thoracic extensor muscles in patients after spinal fusion^35^ or thoracic spine trauma^11^. The age-related changes in posterior extensor muscles described in this study can serve as a reference for changes in thoracic extensor muscles in association with various thoracic spinal disorders or nonsurgical and surgical treatments for these disorders.

## Conclusion

The thoracic posterior extensor muscles of the subjects in this study showed an increase in CSAs over 10 years. Exercise habit influenced the change in CSAs of the erector spinae muscles and the combined extensor muscles. In addition, fat infiltration into the thoracic posterior extensor muscles increased significantly over 10 years. The results of this study could provide useful information for understanding the effect of age on the posterior extensor muscles in the thoracic spine.

## Supplementary Information

Supplementary Information 1.

Supplementary Information 2.
