# Supplementary material for: Changes in cross-sectional areas of posterior extensor muscles in thoracic spine: a 10-year longitudinal MRI study
Source: Sci Rep. 2022 Aug 30;12:14717. doi: 10.1038/s41598-022-19000-2 (PMC9427759; doi:10.1038/s41598-022-19000-2)
Supplement: Supplementary file 1 — Supplementary Information 1. [file 41598_2022_19000_MOESM1_ESM.pdf]

Supplementary 1. Magnetic resonance imaging protocol at each participating institution

Institution	View	Sequence	TE *(ms)*	TR *(ms)*	ETL	NEX	Slice FOV *(%)*	FOV *(cm)*	Phase matrix	Freq. matrix
A	T-spine T2 sag	FSE	100	5,000	24	2	4	24	192	256
	T-spine T1 sag	FSE	Min. full (10)	460	2	2	4	24	192	256
	T-spine T2 ax	FSE	102	5,000	16	2	5	16	192	256
B	T-spine T2 sag	FSE	108	4,000	17	2	4	25	224	320
	T-spine T1 sag	FSE	10	500	7	5	4	25	224	320
	T-spine T2 ax	FSE	90	4,000	23	2	4	14	160	208
C	T-spine T2 sag	FSE	100	5,000	24	2	4	24	208	256
	T-spine T1 sag	FSE	8.2	380	4	2	4	24	208	256
	T-spine T2 ax	FSE	102	5,000	20	2	5	16	224	256
D	T-spine T2 sag	FSE	95	3,000	25	4	4	24	224	320
	T-spine T1 sag	T1 FLAIR	Min. full	1,750	10	3	4	24	192	320
	T-spine T2 ax	FSE	85	2,000	12	4	4	16	192	288
E	T-spine T2 sag	FSE	100	5,000	24	2	4	24	208	256
	T-spine T1 sag	FSE	8.2	380	4	2	4	24	208	256
	T-spine T2 ax	FSE	102	5,000	20	2	5	16	224	256
F	T-spine T2 sag	FSE	100	3,000	26	1	3	24	210	304
	T-spine T1 sag	FSE	15	570	3	1	3	24	152	256
	T-spine T2 ax	FSE	90	4,400	26	1	3.5	16	228	304
G	T-spine T2 sag	FSE	101	5,000	24	2	4	24	224	320
	T-spine T1 sag	FSE	9.2	400	2	2	4	24	192	256
	T-spine T2 ax	FSE	93	5,000	11	2	5	16	192	256
H	T-spine T2 sag	FSE	96	3,000	15	2	4.5	32	256	512
	T-spine T1 sag	FSE	9.2	500	3	2	4.5	32	314	448
	T-spine T2 ax	FSE	93	4,500	14	2	3.5	20	269	384

* echo time; TR, repetition time; ETL, echo train length; NEX, number of excitations; FOV, field of view; freq., frequency; sag, sagittal; ax, axial; FSE, fast-spin-echo; FLAIR, fluid-attenuated inversion recovery; TSE, turbo-spin-echo
