# Supplementary material for: Changes in cross-sectional areas of posterior extensor muscles in thoracic spine: a 10-year longitudinal MRI study
Source: Sci Rep. 2022 Aug 30;12:14717. doi: 10.1038/s41598-022-19000-2 (PMC9427759; doi:10.1038/s41598-022-19000-2)
Supplement: Supplementary file 2 — Supplementary Information 2. [file 41598_2022_19000_MOESM2_ESM.pdf]

Supplement 1. Grading system for magnetic resonance evaluation.

1. Decrease in signal intensity of intervertebral disc
Grade 0: as bright as or slightly lesser bright than the cerebrospinal fluid
Grade 1: markedly darker than the cerebrospinal fluid
Grade 2: no signal
2. Anterior compression of the dura and spinal cord
Grade 0: no compression
Grade 1: compression on the dural sac only
Grade 2: compression on less than one-third of the spinal cord
Grade 3: compression on more than one-third and less than two-third of the spinal cord
Grade 4: compression on more than two-third of the spinal cord
3. Posterior disc protrusion
Grade 0: no protrusion
Grade 1: disc material protruding beyond the posterior margin of the vertebral body without cord compression
Grade 2: beyond the vertebral body with cord compression
4. Disc space narrowing
Grade 0: 100%–75% of the upper healthy disc height
Grade 1: 75%–50% of the upper healthy disc height
Grade 2: <50% of the upper healthy disc height
5. Foraminal stenosis
Grade 0: no stenosis
Grade 1: foraminal stenosis
